# HIF-1α in Osteoarthritis: From Pathogenesis to Therapeutic Implications

**DOI:** 10.3389/fphar.2022.927126

**Published:** 2022-07-05

**Authors:** Chu-Yang Zeng, Xi-Feng Wang, Fu-Zhou Hua

**Affiliations:** ^1^ Department of Anesthesiology, The Second Affiliated Hospital of Nanchang University, Nanchang, China; ^2^ Department of Rehabilitation Medicine, The Third Hospital of Hebei Medical University, Shijiazhuang, China; ^3^ Department of Anesthesiology, The First Affiliated Hospital of Nanchang University, Nanchang, China

**Keywords:** osteoarthritis, HIF-1α, hypoxia, chondrocytes, glycolysis, mitophagy

## Abstract

Osteoarthritis is a common age-related joint degenerative disease. Pain, swelling, brief morning stiffness, and functional limitations are its main characteristics. There are still no well-established strategies to cure osteoarthritis. Therefore, better clarification of mechanisms associated with the onset and progression of osteoarthritis is critical to provide a theoretical basis for the establishment of novel preventive and therapeutic strategies. Chondrocytes exist in a hypoxic environment, and HIF-1α plays a vital role in regulating hypoxic response. HIF-1α responds to cellular oxygenation decreases in tissue regulating survival and growth arrest of chondrocytes. The activation of HIF-1α could regulate autophagy and apoptosis of chondrocytes, decrease inflammatory cytokine synthesis, and regulate the chondrocyte extracellular matrix environment. Moreover, it could maintain the chondrogenic phenotype that regulates glycolysis and the mitochondrial function of osteoarthritis, resulting in a denser collagen matrix that delays cartilage degradation. Thus, HIF-1α is likely to be a crucial therapeutic target for osteoarthritis *via* regulating chondrocyte inflammation and metabolism. In this review, we summarize the mechanism of hypoxia in the pathogenic mechanisms of osteoarthritis, and focus on a series of therapeutic treatments targeting HIF-1α for osteoarthritis. Further clarification of the regulatory mechanisms of HIF-1α in osteoarthritis may provide more useful clues to developing novel osteoarthritis treatment strategies.

## 1 Introduction

Osteoarthritis (OA), the most common reason for joint disease, is an age-related joint degenerative disease and the leading cause of laboral disability in older adults (Guillen et al., 2021). The main clinical characteristics of patients with OA include pain, swelling, brief morning stiffness, and functional limitations. These symptoms will decrease their quality of life and affect their social ability (Hunter and Bierma-Zeinstra, 2019). The data indicated that the knee is the most affected joint of OA, followed by the hand and hip (Bijlsma et al., 2011). The prevalence of symptomatic OA is much higher than radiographic OA, and woman is more than men (Nelson, 2018). In 2021, it was reported that more than 240million people worldwide suffer from OA, including an estimated more than 32 million in the United States (Katz et al., 2021). It is expected to continue with increased life expectancy and the aging of the global population grows by a certain amount every year (Xie et al., 2021). At the end stage of OA, these patients require total joint replacement and rehabilitation to reconstruct joint function, which will carry a huge financial burden on family and country (Benderdour et al., 2015).

OA can be subdivided into primary OA and secondary OA. Genetic factors, aging, mechanical loading, metabolic disorders, hormonal alterations are mainly risks of primary OA (Zheng et al., 2021). Secondary OA is caused by underlying predisposing factors, such as trauma and surgery (Taruc-Uy and Lynch, 2013). Most degenerative OA belongs to primary OA and involves the whole joint. The main pathological changes include cartilage erosion, synovial inflammation, and subchondral sclerosis with osteophyte formation (Salgado et al., 2020). Clinically, the main treatments for degenerative OA are pharmacotherapy, physiotherapy, surgery, and rehabilitation (Sharma, 2021), such as nonsteroidal anti-inflammatory drugs, extracorporeal shock wave therapy, total knee replacement and strengthening exercise (Sharma, 2021). In addition, recent research found that telomere shortening may through inflammation contribute to OA, which could be an epigenetic factor of OA in the future (Fragkiadaki et al., 2020). However, the most effective treatments for degenerative OA will be drugs targeting the conditions that accelerate disease progression rather than the chondroprotective agents preventing the initiation of damage (Burr and Gallant, 2012).

In recent years, more and more evidence has shown that inflammation takes center stage in the pathogenesis of degenerative OA. Damaged chondrocytes, under the stimulation of pro-inflammatory cytokines (IL-6, IL-8, and TNF-α), nitric oxide (NO), as well as prostaglandins exhibit a transient proliferative response and increased matrix synthesis, such as matrix metalloproteinases (MMPs), collagen II, aggrecan, proteoglycan (Boulestreau et al., 2021). In addition, two kinds of major enzymes: MMPs and ADAMTSs (a disintegrin and a metalloprotease with thrombospondin motifs), are implicated in the degradation of a matrix in the degenerative cartilage (Wang and He, 2018; Yunus et al., 2020). The continuous production of proteases driven by proinflammatory cytokines leads to extensive matrix degradation and loss which stimulates chondrocytes to produce more cytokines and proteases in an autocrine and paracrine manner (Yunus et al., 2020). At the early stages of degenerative OA, the main change is the altered molecular composition and organization in the extracellular matrix. During the progressive stages of degenerative OA, severe inflammation leads to the gradual deterioration of articular cartilage, thickening of the subchondral plate, and osteophyte formation (Xia et al., 2014).

Hypoxia-inducible factor-1 α (HIF-1α) is a highly conserved transcription factor that responds to cellular oxygenation decreases in tissue (Warbrick and Rabkin, 2019). In hypoxia cells, HIF-1α induces the switch from oxidative to glycolytic metabolism, which involves changes in the expression of encoding metabolic enzymes, transporters, and mitochondrial proteins involved in glucose metabolism (Semenza, 2014). Moreover, HIF-1α plays a key role in increasing the synthesis of the key pro-inflammatory cytokine IL-1β (Scholz and Taylor, 2013). Catabolic stresses, IL-1β, and oxidative stress induce the expression of HIF-1α in chondrocytes (Yudoh et al., 2005). The accumulation of the HIF-1α protein was detected as early as 4 h after initiation of treatment with IL-1β and correlated with the concentration and exposure time with treatment of IL-1β. The treatment of OA and decreasing IL-1β in chondrocytes can significantly downregulate HIF-1α gene expression (Malkov et al., 2021). HIF-1α expression was explicitly observed in mature and hypertrophic chondrocytes, and the activity of HIF-1α was enhanced (Shirakura et al., 2010). In hypoxia condition, OA chondrocytes had significantly lower expression of MMPs and ADAMTSs (Markway et al., 2013). Therefore, HIF-1α plays a vital role in the pathological mechanism of OA, suggesting that HIF-1α may be an effective therapeutic target for degenerative OA.

In this review, we explore knowledge regarding the activation of the HIF-1α and its pivotal role in pathogenic mechanisms of degenerative OA. In addition, we introduce several OA treatments that target the HIF-1α pathway. We focus on the four aspects of molecular mechanisms of HIF-1α and OA: the regulation and function of HIF-1α; the relationship between hypoxia and OA; the important signal pathway for HIF-1α and OA; application of therapies targeting the HIF-1α in the treatment of OA. By summarizing recent advances in research to characterize the relationship between OA and survival and growth of chondrocytes, and the potential involvement of HIF-1α transcription factors in the disease process, we highlight avenues for further research and potential therapeutic targets for this disease.

We used “osteoarthritis,” “HIF-1α,” “hypoxia,” “mechanism,” “inflammation,” and “treatment” as keywords. Then we searched PubMed, CINAHL, and the web of science for methodological papers on articles from July 1988 to April 2022, especially recently 5 years. Having examined 923 full articles, we finally selected 230 articles for this review.

## 2 Regulation and Function of HIF-1 α

### 2.1 Structure of HIF-1α Protein

**FIGURE 1:**
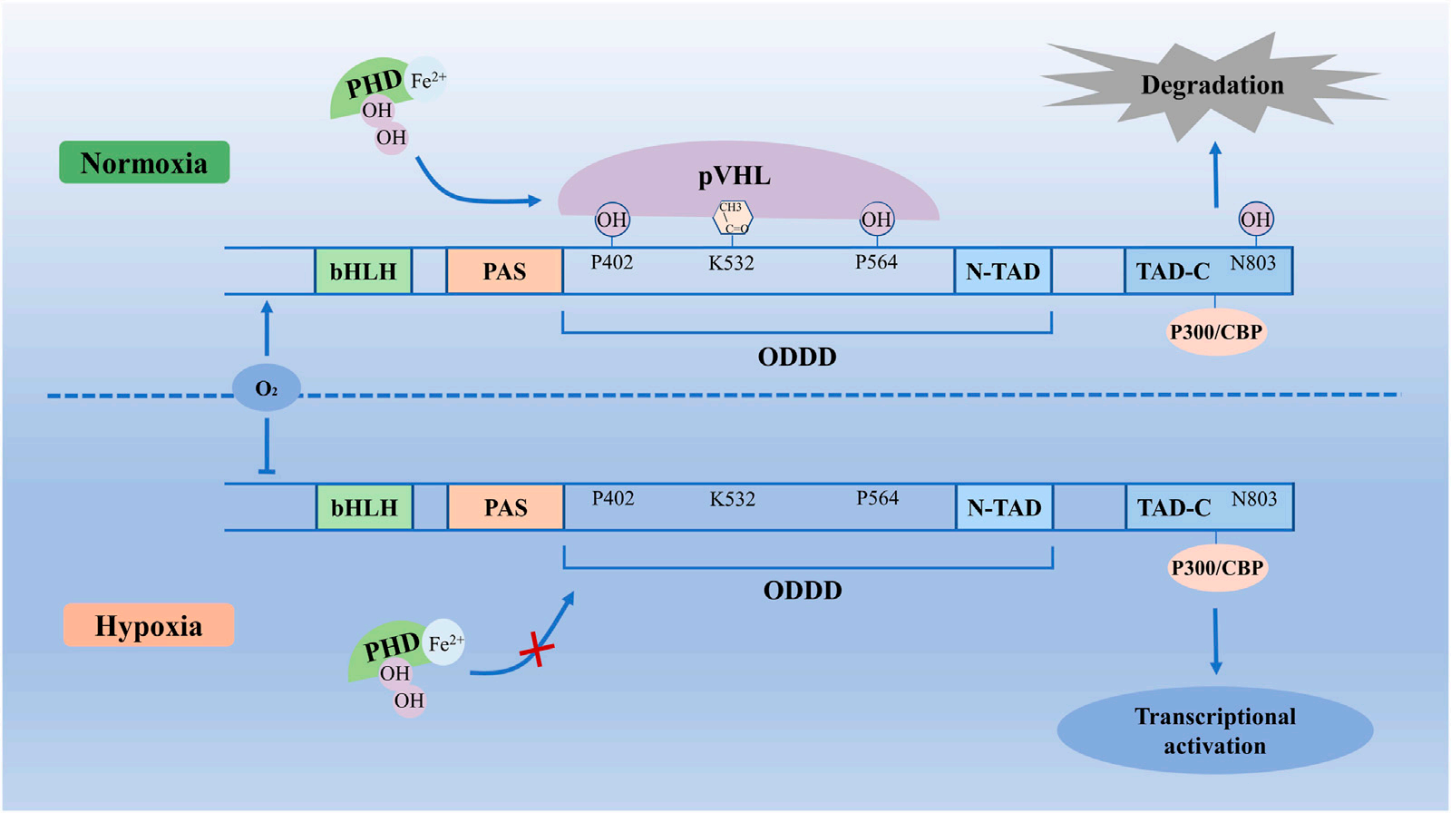
Molecular mechanisms of HIF-1α degradation and stability. In normoxic conditions, with Fe^2+^ and 2-oxoglutarate, HIF-1α is hydroxylated by PHDs on Pro402 and Pro564, which allows the HIF-1α recognition by pVHL. Then HIF-1α protein is subject to rapid degradation by the pVHL mediated ubiquitin-proteasome pathway. In hypoxic conditions, due to the decreased activity of PHDs and inhibited hydroxylation and inactivation of pVHL, HIF-1α escapes proteasomal degradation. Stabilized HIF-1α binds with p300/CBP to increase transcriptional activity, which increases HIF-1α protein stability and thereby regulates transcriptional activation. bHLH, basic helix–loop–helix; HIF-1α, Hypoxia-inducible factor-1 α; ODDD, oxygen-dependent degradation domain; PAS, Per-ARNT-Sim homology; PHD, prolyl hydroxylase; pVHL, von Hippel-Lindau tumor suppressor protein; TAD-N, N-terminal transactivation domains; TAD-C, C-terminal transactivation domains.

HIF-1α is a transcriptional factor encoded by the HIF-1α gene located within chromosome 14q21-24, which consists of 826 amino acids, and was regulated by hypoxia signal (Fernandez-Torres et al., 2017a). HIF-1α has an oxygen-dependent degradation domain (ODDD), which contains two sites for hydroxylation, Pro 402 and Pro 564, and each site contains a conserved LXXLAP motif (Ke and Costa, 2006). ODDD is located in the central region of HIF-1α, which is the main domain to mediate oxygen-regulated stability (Bruick and McKnight, 2001). In addition, the HIF-1α subunit contains TAD-N and TAD-C (N- terminal transactivation domains and C-terminal transactivation domains) bridged by an inhibitory domain (Ruas et al., 2002). The TAD-N overlaps with the ODDD and is continuous with protein stability. The TAD-C interacts with coactivators such as p300/CBP, independent of protein stability (Lee et al., 2004). In normoxic conditions, Fe^2+^ and 2-oxoglutarate as co-factors, HIF-1α is hydroxylated by a family of prolyl-hydroxylase (PHD) on Pro402 and Pro564 (Fernandez-Torres et al., 2017a). This hydroxylation process allows the hydroxylated HIF-1α recognition by the von Hippel-Lindau tumor suppressor protein (pVHL). Then HIF-1α protein becomes ubiquitylated and is subject to rapid degradation by the process of pVHL mediated ubiquitin-proteasome pathway (Lee et al., 2004). In hypoxic conditions, due to the decreased activity of PHDs inhibiting hydroxylation and inactivation of pVHL, HIF-1α escapes proteasomal degradation (Yang et al., 2019b). Stabilized HIF-1α binds with p300/CBP, steroid receptor coactivator-1 family coactivators, nuclear redox regulator Ref-1, and molecular chaperon heat shock protein 90 to increase transcriptional activity (Weidemann and Johnson, 2008), which will increase HIF-1α protein stability and thereby regulate the adaptive cellular response to hypoxia (Figure 1). However, when hypoxic cells reoxygenate, accumulated HIF-1α degrades rapidly, and the rate of HIF-1α degradation depends on the duration of hypoxic stress (Berra et al., 2001).

The central role of HIF-1α in hypoxia-induced transcription is involved in various cellular processes, such as embryonic development, metabolism, cell proliferation, inflammation, and angiogenesis (Marmorstein and Simon, 2015). It could regulate macrophage survival and differentiation, neutrophil survival, T-cell differentiation, and dendritic cell function (Scholz and Taylor, 2013). HIF-1α regulates the hypoxic response by controlling genes with metabolic functions, such as glucose transport and metabolism, angiogenic factors like vascular endothelial-derived growth factor (VEGF), proteins of the cell cycle, and apoptotic machinery (Schipani, 2005). Analysis of knockout mice has shown that HIF-1α is required for embryonic development and survival (Semenza, 2001). A study showed that HIF-1α deficient mouse embryos arrest in their gestation development and die with severe cardiovascular, massive cell death and neural tube defects (Iyer et al., 1998). In addition, HIF-1α deficiency significantly altered the quality and quantity of mouse embryonic stem cells derived cardiomyocytes (Kudova et al., 2016), decreased the aggrecan and type-II collagen in nucleus pulposus (Wu et al., 2013), reduced the population of chondrocytes in the growth plates and affected endochondral ossification (Hong et al., 2021).

### 2.2 Regulation of HIF-1α in Diseases

**FIGURE 2:**
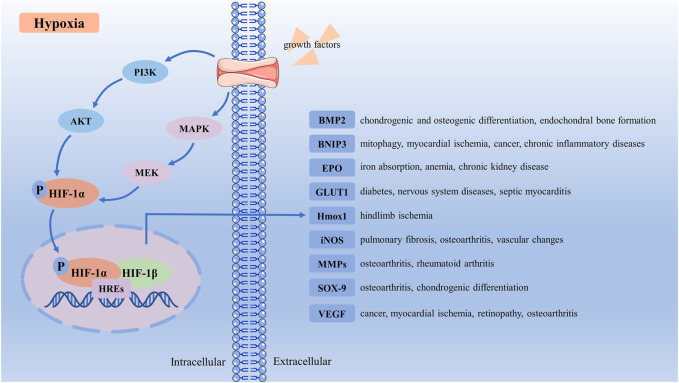
Schematic representation of HIF-1α activation signaling pathway. Under hypoxia conditions, growth factors bind to receptor tyrosine kinase, and activate the PI3K and MAPK pathways. Then PI3K and MAPK activate their downstream AKT and MEK, which phosphorylate HIF-1α. Phosphorylated HIF-1α translocates to the nucleus, where it binds to HIF-1β and HREs to form the [(HIF-1α + HIF-1β) + HREs] complex. This functionally active transcriptional complex promotes regulation and expression of HIF-dependent adaptive genes, upregulates HIF-1α activity, and regulates many target receptors, such as BMP2, BNIP3, EPO, GLUT1, HMOX-1, iNOS, MMPs, SOX9, and VEGF. BMP2, bone morphogenetic protein 2; BNIP3, BCL2 and adenovirus E1B 19-kDa-interacting protein 3; EPO, erythropoietin; GLUT1, glucose transporter 1; HIF-1α, Hypoxia-inducible factor-1α; HMOX-1, heme oxygenase-1; HREs, hypoxia response elements; iNOS, inducible nitric oxide synthase; MMPs, matrix metalloproteinases; VEGF, vascular endothelial-derived growth factor.

Growth factors induce HIF-1α protein translation of hypoxia, and hypoxia is associated with decreased degradation of HIF-1α (Lee et al., 2004). It has been well characterized that PI3K/AKT pathway and MAPK pathway are central downstream signaling pathways of growth factors (Zhan et al., 2015). Growth factors bind to receptor tyrosine kinase and activate the PI3K and MAPK pathways (Lee et al., 2004). Then, PI3K activates its downstream AKT and PKA and positively regulates HIF-1α at the protein level (Zhang et al., 2018c). In the MAPK pathway, growth factors activate RAS and its downstream, MAPK and MEK. The MAPK has been correlated with TAD-C activity as enhanced activation of MAPK increases HIF-1α-mediated transactivation (Lisy and Peet, 2008). These two signal pathways could phosphorylate HIF-1α *in vitro* and *vivo* (Richard et al., 1999; Kietzmann et al., 2016). Under conditions of hypoxia, PHDs activity decreases, which can stabilize HIF-1α and accumulate in the cytoplasm to be phosphorylated by MAPK or PI3K (Fan et al., 2014). When oxygen levels were lower than 6%, phosphorylated HIF-1α could migrate to the nucleus where it binds to HIF-1β to form the complex (HIF-1α + HIF-1β). Then complex binds to hypoxia response elements (HREs) to form the [(HIF-1α + HIF-1β) + HREs] complex (Losso and Bawadi, 2005), which will initiate transcription and upregulate HIF-1α activity (Kobayashi et al., 2017). This functionally active transcriptional complex promotes the regulation and expression of HIF-dependent adaptive genes, such as bone morphogenetic protein 2 (BMP2), BCL2 and adenovirus E1B 19-kDa-interacting protein 3 (BNIP3), erythropoietin (EPO), glucose transporter 1 (GLUT1), heme oxygenase-1 (HMOX-1), inducible nitric oxide synthase (iNOS), MMPs, SOX-9, VEGF (Patra et al., 2019) (Figure 2).

BMP2 is one of the main chondrogenic growth factors involved in cartilage regeneration (Zhou et al., 2016). HIF-1α could regulate BMP-induced chondrogenic differentiation, osteogenic differentiation, and endochondral bone formation (Zhou et al., 2015). BNIP3 is a protein with homology to BCL2 in the BH3 domain, inducing cell death and autophagy (Zhang and Ney, 2009). HIF-1α/BNIP3 mediated mitophagy is an important pathway for treating myocardial ischemia, cancer, and chronic inflammatory diseases (Zhang et al., 2019b). HIF-1α regulates EPO production in the kidney. Inhibiting HIF-PHD in chronic kidney disease could lead to increased EPO production and better iron absorption, ameliorating anemia (Voit and Sankaran, 2020). GLUT1 location at the blood-brain barrier mainly regulates diabetes and nervous system diseases (Koch and Weber, 2019). Hypoxia enhances the expression of HIF-1α, prolongs GLUTI gene expression, and protects septic cardiomyocytes (Bateman et al., 2007). As a downstream target of the transcription factor HIF-1α, Hmox1 could promote effective glucose utilization to limit ischemia-induced tissue necrosis and autoamputations in skeletal muscle (Dunn et al., 2021). Pulmonary fibrosis cellular and vascular changes in the retina are correlated with impaired iNOS (Miller et al., 2013; Kseibati et al., 2020). HIF-1α contributes to the induction of iNOS under hypoxia, and the expression of iNOS could inhibit the activity of HIF-1α (Yang et al., 2002; Wilmes et al., 2020). MMPs, cleaves, and rebuild connective tissue components mediate inflammation in OA and rheumatoid arthritis (Zitka et al., 2010). The hypoxia-mediated differential change of MMP-1 and MMP-13 expression in IL-1β-stimulated arthropathies depended on HIF-1α expression (Lee et al., 2012b). As the key transcription factor controlling collagen II and aggrecan expression, SOX-9 could promote chondrocyte differentiation and proliferation (Lafont, 2010). Hypoxia upregulates SOX-9 expression in chondrocytes in a HIF-1α-dependent manner (Li et al., 2018). VEGF, vital in vasculogenesis and angiogenesis, has been demonstrated to be important in the molecular pathogenesis of tumor growth and metastasis related to myocardial ischemia and retinopathy associated with several blinding eye diseases (Apte et al., 2019). The increase of HIF-1α promotes VEGF expression and blood vessel production (Zhang et al., 2018a).

Currently, researchers have concentrated on the role of HIF-1α in numerous diseases. HIF-1α plays an important role in several diseases, such as cancer, cardiovascular disease, nephropathy, mood disorder, and musculoskeletal disorders. Moreover, regulating the expression and activity of HIF-1α exhibits potent therapeutic effects in those diseases. In chronic inflammatory diseases, HIF-1α stabilization helps the host apparent prolonged mycobacterial infection *via* neutrophil activation (Schild et al., 2020). Yudoh et al. (2005) found that HIF-1α can protect chondrocytes from cell death induced by pro-inflammatory cytokines. Furthermore, HIF-1α could regulate chondrogenesis by regulating SOX-9 expression at the genetic level and also regulate both autophagy and apoptosis of OA chondrocytes (Zhang et al., 2015b). Therefore, it is important to summarize the mechanisms of the HIF-1α in OA and potential treatments targeting the HIF-1α in OA.

## 3 Evidence for Hypoxia and Osteoarthritis

### 3.1 Hypoxia and Articular Cartilage

Due to the microstructure of the joint, chondrocytes have existed in a hypoxic environment for a long time (Yudoh et al., 2005). Cartilage is relatively thick, and it is estimated that chondrocytes of the joint surface expose to approximately 6%–10% oxygen, whereas chondrocytes from the deepest layers of the articular cartilage may have access to only 1%–6% oxygen in cartilage (Kiaer et al., 1988). Oxygen is a critical parameter proposed to modulate chondrocyte metabolic activity (Strobel et al., 2010). The majority of the chondrocyte’s energy requirements come from glycolysis, with little energy from oxidative phosphorylation (Archer and Francis-West, 2003). As oxygen levels dropped, O_2_-dependent mitochondrial oxidative phosphorylation was inhibited, contributing to the fall in glycolysis and ATP. Hence, matrix synthesis is less (Kierans and Taylor, 2021). Matrix of articular cartilage consists largely of collagen (especially collagen type II) and the large aggregating proteoglycan, aggrecan, containing poly-sulphated side chains, which are avascular, hyperosmotic acidic (Gibson et al., 2008). Compared with oxygen level at 20%, 2% oxygen level increases collagen I and II content, tensile modulus, and compressive properties (Schipani, 2010). The mature chondrocytes are more sensitive to hypoxia, and higher-level hypoxia can induce endoplasmic reticulum stress, followed by chondrocytes apoptosis (Huang et al., 2017).

### 3.2 Hypoxia and Osteoarthritis

Hypoxia and inflammation persistently exist in the pathological progress of knee OA (Zhang et al., 2019a). In the late stage of OA, due to increased oxygen usage by the synovial membrane of OA, the joint cartilage cells are partially deprived of their oxygen supply (Svalastoga and Kiœr, 1989). The concentrations of oxygen within the cartilage matrix gradually diminish with increasing proximity to the calcified cartilage layer, and this is particularly relevant in mature cartilage (Mobasheri et al., 2002). In addition, hypoxia upregulated genes associated with chondrocyte differentiation and induced phenotypic changes consistent with chondrocyte lineage progression (Robins et al., 2005). Synovial fluids from joints of patients with OA have less oxygen than synovial fluids from healthy joints. Furthermore, synovitis could drive the extent of angiogenesis and inflammation under low oxygen tensions (Bonnet, 2005). In the tissue of patients with OA, both cartilage and subchondral bone show signs of hypoxia, compared to normal bone obtained postmortem (Chang et al., 2014). Low oxygen will enhance *in vivo* chondrogenesis of rat mesenchymal stem cells (Murphy et al., 2009). It may delay or inhibit terminal differentiation of mesenchymal stem cells through induction and maintenance of SOX9 levels (Murphy et al., 2009). Besides, in patients with OA, reduced oxygen levels in synovial fluids will accelerate the posttranslational modification of collagen type II, thus contributing to the increased synthesis of collagen type II during OA (Pfander and Gelse, 2007). Hypoxia also increased the expression of cyclooxygenase-2 and the production of prostaglandin E2 by OA osteoblasts, which could exacerbate the progression of OA (Chang et al., 2014). However, severe hypoxia may kill chondrocytes, at least in part, by altering post-translational modifications of collagen (Yao et al., 2020). Therefore, articular cartilage hypoxia could be a potential target for OA treatments.

## 4 Pathogenesis of HIF-1 α in Osteoarthritis

During cartilage energy, generation, and matrix synthesis of chondrocytes in healthy development nor osteoarthritic cartilage, HIF-1α has pivotal importance for the survival and growth arrest of chondrocytes (Pfander et al., 2006). Such as the regulation of anaerobic energy generation, glucose transport, and matrix synthesis by articular chondrocytes could support the metabolic requirements of proliferation following hypoxic stimulation (Gabriely et al., 2017). In normal and OA human chondrocytes under normoxic and hypoxic conditions, an easily detectable level of HIF-1α protein expression could be observed (Coimbra et al., 2004). In OA cartilage samples, there are increased number of chondrocytes staining for the transcription factor HIF-1α and its target genes (Grimmer et al., 2006). Furthermore, there are the lowest oxygen levels in deeper layers of cartilage, where HIF-1α is tonic active and accumulated within the chondrocyte (Pfander and Gelse, 2007). The present findings showed that patients with more severe knee OA had marked higher levels of HIF-1α in their articular cartilage and synovial fluid (Qing et al., 2017).

### 4.1 HIF-1α and Chondrocytes

HIF-1α is extensively localized in chondrocytes from the earlier differentiation stages and induces initial chondrogenesis, joint development, cartilage matrix synthesis, and cell survival (Saito and Kawaguchi, 2010). The HIF-1α knockout rats have a massive cell death in the center of the cartilaginous elements and a slight delay in the process of chondrocyte differentiation at its periphery (Schipani et al., 2001). During hypoxic and normoxic conditions, functional inactivation of HIF-1α in IL-1β-induced OA resulted in a significantly increased number of apoptotic chondrocytes (Pfander et al., 2006). Moreover, HIF-1α could protect articular cartilage by promoting chondrocyte phenotype, maintaining chondrocyte viability, and supporting metabolic adaptation in hypoxic conditions (Zhang et al., 2015b). The stabilization of HIF-1α enhances the chondrogenic differentiation of progenitor cells and minimizes chondrocyte hypertrophy (Taheem et al., 2020). Chondrocytes are differentiated from mesenchymal stem cells (Yao et al., 2020). Hypoxia by activating SOX-9 *via* HIF-1α promotes the differentiation of mesenchymal stem cells along a chondrocyte pathway, which is required for chondrocyte differentiation and cartilage formation (Robins et al., 2005; Fernandez-Torres et al., 2017b). In PHD-deficient chondrocytes, HIF-1α induced metabolic changes could reduce collagen synthesis and enhance collagen modifications, resulting in a denser collagen matrix that hinders cartilage degradation (Stegen et al., 2019). In PHD2-deficient chondrocytes, decreasing HIF-1α levels was sufficient to reverse the increased hydroxyproline levels, which could prevent the accumulation of collagen remnants and normalize bone mass in an ectopic model of endochondral ossification (van Gastel et al., 2014).

Collagen type II is produced in cartilage by proliferating chondrocytes and the upper hypertrophic chondrocytes (Schipani, 2010). Collagen type II degradation in chondrocytes is represented a key characteristic of OA (Bo et al., 2018). Delivery of a HIF-1α expression vector could increase a panel of chondrogenic transcription factors and enhance the expression of genes for both collagen type II and the proteoglycan aggrecan (Taheem et al., 2020). In PHD2-deficient chondrocytes, the collagen synthesis decreased and the total and extracellular collagen type II content increased (Stegen et al., 2019). HIF-1α knock-out strongly decreases aggrecan and type II collagen expressions in murine embryos and chondrocytes (Duval et al., 2012). In hypoxia conditions, HIF-1α could maintain anaerobic glycolysis and thereby extracellular matrix synthesis, including enhancing collagen type II and aggrecan of epiphyseal chondrocytes (Pfander et al., 2003). Increasing the expression of HIF-1α with cobalt chloride in a normoxic environment effectively promoted the expression of SOX-9, collagen II chain, and aggrecan in chondrocytes, which was beneficial in promoting the maintenance of the chondrogenic phenotype (Li et al., 2018).

### 4.2 HIF-1α and Molecular Composition of Extracellular Matrix

At the early stages of KOA, the molecular composition and organization in the extracellular matrix have altered first. The activity of HIF-1α could regulate extracellular matrix synthesis, mainly about anaerobic energy generation, proteoglycan synthesis, and maintain cartilage homeostasis of articular chondrocytes (Yudoh et al., 2005). In less partial oxygen pressure and higher mechanical stress, chondrocytes in OA will produce excess mitochondrial reactive oxygen species (ROS) (Kulkarni et al., 2021), which favor neo-cartilage hyaline matrix formation (Ollitrault et al., 2015). The accumulation of HIF-1α is responsible for the increased matrix deposition in the growth plate (Weng et al., 2014), such as type II collagen and MMPs (Pfander et al., 2004). Hypoxia could maintain low Wnt/β-catenin signaling *via* HIF-1α, which decreased suppression of ADAMTS-5 and MMP-13, then prevented chondrocyte catabolism (Thoms et al., 2013; Bouaziz et al., 2016). The activation of HIF-1α could promote the secretion of the extracellular matrix and elevate the expression of genes including SOX-9, type II collagen and aggrecan (Wang et al., 2020c). In addition, increased HIF-1α could aggravate synovial fibrosis via fibroblast-like synoviocytes pyroptosis in rats with knee OA (Zhang et al., 2019a).

### 4.3 HIF-1α and Autophagy of Chondrocytes

Autophagy, a lysosomal degradation pathway, is an important cellular event during chondrocyte development, which plays both a cytoprotective and death-promoting role in the pathogenesis of OA (Chang et al., 2013). Basal levels of mitophagy maintain cellular homeostasis and protect cells (Hu et al., 2020b). It has been demonstrated that HIF-1α regulates autophagy inactivation under inflammatory stress (Malladi et al., 2007). Lu et al. (2021) found that in a HIF-1α elevated environment, the chondroprotective effect of autophagy is increased. Under moderately hypoxic conditions, cells secreted more IL-1β (Zhang et al., 2018b), and IL-1β inhibits rat chondrocytes’ cell cycle and proliferation rate and reduces the autophagy rate (Xue et al., 2017). The mechanisms of HIF-1α induced autophagy to contain modulation of the beclin-1/Bcl-2 complex (Hu et al., 2020b). HIF-1α stabilization will promote hypoxia-induced apoptosis *via* mitophagy, reducing the levels of reactive oxygen species generated and recovering extracellular matrix metabolic unbalance in the chondrocytes, which could ameliorate cartilage degradation in the mice OA model (Hu et al., 2020b). Moreover, HIF-1α promotes chondrocytes apoptosis and autophagy *via* depressing Bcl-2, modulating the autophagic proteins and caspase-8 (Zhang et al., 2015a).

### 4.4 HIF-1α and Glycolysis of Chondrocytes

Fetal chondrocytes are highly glycolytic cells. Articular chondrocytes appear to show a negative Pasteur effect, whereby glycolysis falls as O_2_ levels drop, contributing to the fall in ATP (Lee and Urban, 1997). Marcus found that the glycolytic rates of chondrocytes greatly exceeded those of HeLa cells grown under similar concentrations (Marcus, 1973). OA chondrocytes, which are metabolically activated, rely on HIF-1 to instigate anaerobic ATP generation via increased glucose uptake and utilization in order to compensate for the accelerated energy consumption during OA (Pfander et al., 2006). It is established that HIF-1α promotes glycolysis and lactate fermentation whereas it suppresses mitochondrial respiration by modulating either directly or indirectly a variety of genes involved in bioenergetics (Yao et al., 2020). HIF-1α also augments the expression of glycolytic enzymes such as phosphoglycerate kinase, which converts 1,3-diphosphoglycerate to 3-phosphoglycerate during glycolysis (Semenza et al., 1994). The [(HIF-1α + HIF-1β) + HREs] complex could increase the expression of GLUT1, glucose-6-phosphate dehydrogenase (G6PD), phosphoglycerate kinase 1 (PFK1), and pyruvate dehydrogenase kinase 1 (PDK1), which promotes glucose transfer and anaerobic glycolysis (Wang et al., 2020b). In chondrocytes, Bone Gla Protein decreases the expression of key gluconeogenesis enzymes-phosphoenolpyruvate carboxykinase (PEP) and G6PD, in a HIF-1α–dependent manner (Idelevich et al., 2011). Hypoxia through transcription factor HIF-1α induces the expression of VEGF and EPO and promotes glycolysis. Both could increase the delivery of oxygen and nutrients, together with metabolic adaptations, thereby preventing chondrocyte cell death in the growth plate (Stegen and Carmeliet, 2019).

### 4.5 HIF-1α and Mitochondrial Dysfunction of Chondrocytes

Recent *ex vivo* studies have reported that mitochondrial dysfunction could act as a pathogenic factor in degenerative cartilage disease (Holzer et al., 2019). The pro-survival role of HIF-1α in chondrocytes might be in part mediated through the suppression of oxygen consumption by mitochondria (Long, 2019). Stimulating chondrocyte mitochondrial respiration has a profound impact on the production and consumption of cellular oxygen species, which results in the maintenance of redox homeostasis (Lane et al., 2015). Mitophagy consists of the elimination of depolarized and damaged mitochondria, and the activation of this process could protect against mitochondrial dysfunction, prevent ROS production and improve chondrocyte survival under pathological conditions (Fernandez-Moreno et al., 2022). Targeting the mitophagy pathway in marrow mesenchymal stem cells may improve their survival capacity and chondrogenic differentiation for cell-based therapies in OA (Sun et al., 2021). Overexpression of HIF-1α, through its downstream marker BNIP3, increases mitophagy induced by hypoxia and protects bone cells from apoptosis (Xu et al., 2021). By enhancing BNIP3 expression, HIF-1α could alleviate hypoxia-induced apoptosis, senescence and matrix degradation in chondrocytes through mitophagy (Hu et al., 2020b). In addition, HIF-1α regulates the induction of autophagy and the activity of mitochondrion by regulating uncoupling proteins-3 (UCP-3) expression in chondrocytes, which suppresses treatment sensitivity that promotes chondrocyte deletion from the growth plate (Watanabe et al., 2008).

**FIGURE 3:**
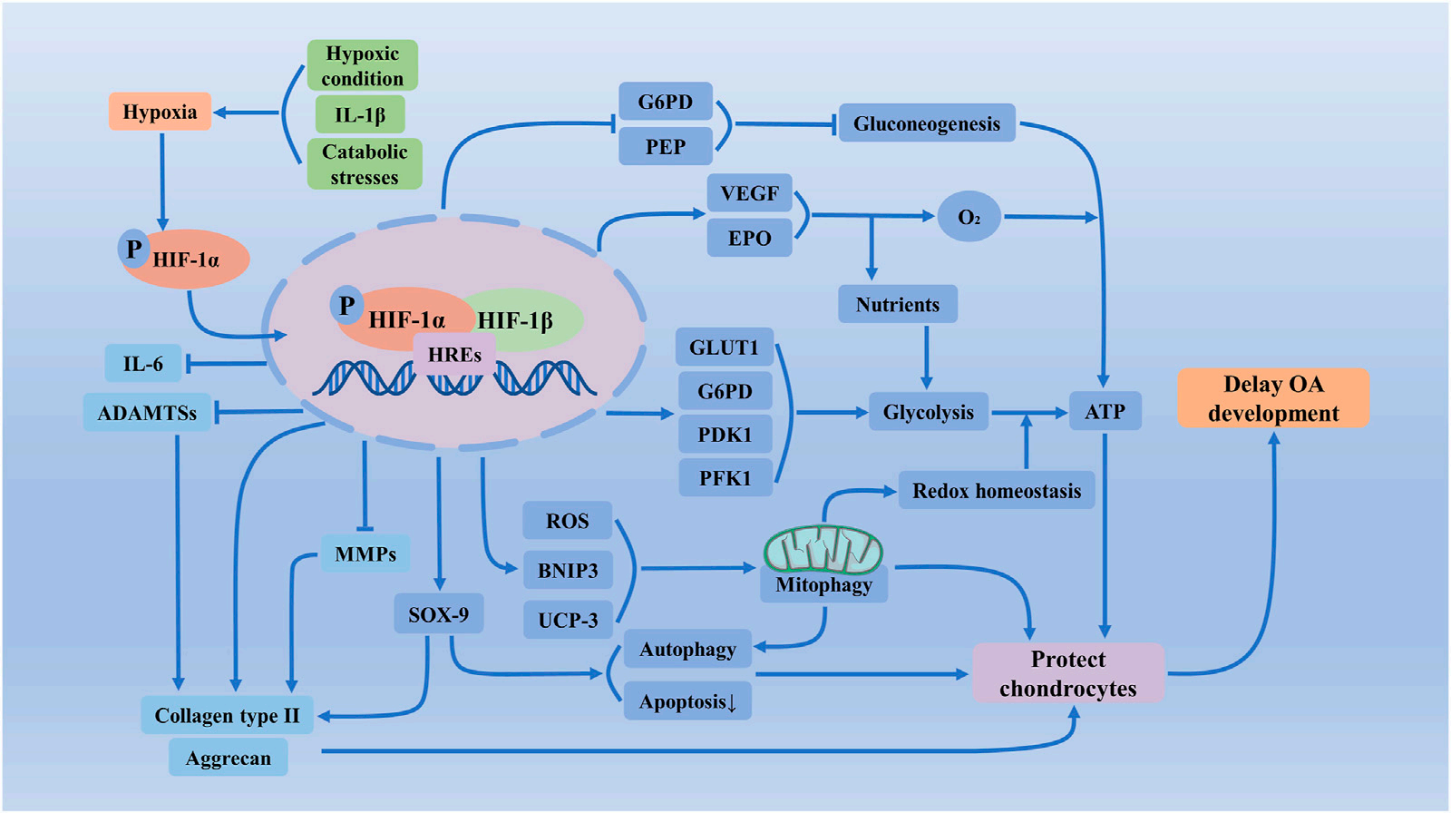
Regulatory mechanisms and crucial influence of HIF-1α and hypoxia signaling on chondrocytes of OA. In less partial oxygen pressure, higher mechanical stress, and inflammation could exacerbate chondrocyte hypoxia, which will accumulate HIF-1α and increase transcriptional activity. Activated HIF-1α via regulating the apoptosis and autophagy of chondrocytes, the synthesis of extracellular matrix, glycolysis, mitochondrial function, and redox reactions of chondrocytes, maintains chondrogenic phenotype, protects articular cartilage and effectively alleviates the development of OA. ADAMTSs, a disintegrin and a metalloprotease with thrombospondin motifs; ATP, adenosine triphosphate; BNIP3, BCL2 and adenovirus E1B 19-kDa-interacting protein 3; EPO, erythropoietin; G6PD, glucose-6-phosphate dehydrogenase; GLUT1, glucose transporter 1; HIF-1α, Hypoxia-inducible factor-1 α; HREs, hypoxia response elements; IL-6, Interleukin-6; MMPs, matrix metalloproteinases; PEP, phosphoenolpyruvate carboxykinase; PDK1, pyruvate dehydrogenase kinase 1; PFK1, phosphoglycerate kinase 1; ROS, reactive oxygen species; UCP-3, uncoupling proteins-3; VEGF, vascular endothelial-derived growth factor.

Normal articular cartilage is hypoxic, and chondrocytes have a specific and adapted response to a low oxygen environment. OA articular cartilage has lower oxygen, which will lead to the accumulation and stabilization of HIF-1α. As shown above, the regulatory mechanisms and crucial influence of HIF-1α and hypoxia signaling on chondrocytes is a new potential target for OA treatment (Figure 3).

## 5 The Important Signal Pathway With HIF-1 α

### 5.1 JAK2/STAT3

The JAK2/STAT3 signaling pathway, a basic and well-conserved intracellular signaling cascade, can be phosphorylated under extracellular stimulation (Zhong et al., 2021). BMP2 inducing tyrosine kinase signaling by JAK2 phosphorylation increases the expression of HIF-1α (Belmokhtar et al., 2011). Hypoxia-induced HIF-1α was inhibited by STAT3 inhibitor, suggesting that the activation of STAT3 signal-regulated HIF-1α expression in the experimental hypoxia (Yang et al., 2022). Recently, more and more evidence has demonstrated that the JAK2/STAT3 signaling pathway has a therapeutic effect on OA progression (Zou et al., 2019). The JAK2/STAT3 signaling pathway is involved in regulating cellular responses to inflammatory factors, including IL-1, IL-6, and MMPs (Luan et al., 2012). Zou et al. (2019) found that the JAK2/STAT3 signaling pathway could regulate chondrocyte metabolism and enhance oxidative stress in OA mouse models. Danshen could decrease the levels of phosphorylated JAK2 and phosphorylated STAT3 in OA cartilage (Xu et al., 2018). HIF-1α induces hypoxic bone mesenchymal stem cells to release small extracellular vesicles, which promote the proliferation, migration, and apoptosis inhibition of chondrocytes through the JAK2/STAT3 signaling pathway (Rong et al., 2021). Furthermore, through JAK2/STAT3 signaling pathways, dopamine can reverse IL-1β-treated NF-κB activation and suppress the cartilage matrix degradation in OA model rats (Lu et al., 2019). By activating JAK2/STAT3 pathway, HIF-1α could promote the expression of RANKL in osteocytes and enhance osteocyte-mediated osteoclastic differentiation *in vitro* (Zhu et al., 2019). Therefore, the JAK2/STAT3 signaling pathway plays a vital role in the pathophysiological process of OA.

### 5.2 Mitogen-Activated Protein Kinases

Mitogen-activated protein kinases (MAPK) belong to a large family of serine-threonine kinases, forming major cell-proliferation signal pathways from the cell surface to the nucleus (Dong et al., 2002). MAPK signal pathway can activate transcription factors HIF-1α with cytoplasmic serine phosphorylation and proteolysis connected to other signal pathways. These pathways include the NF-κB family, Wnt signaling pathway, and Notch signaling pathway (Hommes et al., 2003). MAPK signal transduction pathways could be novel anti-inflammatory targets for the inflammatory response in eukaryotic cells (Hommes et al., 2003). MAPK signaling is implicated in modulating the polarization of M1 macrophages, which could regulate the production of inflammatory cytokines (Zhou et al., 2019a). Imperatorin could suppress inducible NOS by inhibiting the IL-1β induced activation of the ERK-MAPK signaling pathway, reducing collagen-induced OA by reducing synovial hyperplasia (Ahmad et al., 2020). p38/MAPK signaling pathway mediated the hypoxia-induced VEGF production in chondrocytes, whereas JNK was involved in the IL-1β-induced VEGF production (Murata et al., 2006). Moreover, in the chondrocyte’s growth plate, the chondrocyte uses the HIF-1α signaling pathway *via* regulating the VEGF expression to promote angiogenesis and protect cartilage (Choi et al., 2011).

### 5.3 The Nuclear Factor Kappa B

The nuclear factor kappa B (NF-κB) family of transcription factors is a crucial regulator of immune development, immune responses, inflammation, and cancer (Mitchell et al., 2016). NF-κB signal pathway has been verified to be related to numerous skeletal diseases, such as inflammatory arthritis, OA, osteoporosis, and bone metastasis of tumors (Novack, 2011). Especially, NF-κB has become a therapeutic target for OA (Choi et al., 2019). Currently, researchers found that NF-κB signaling affects cartilage matrix remodeling, synovial inflammation, and chondrocyte apoptosis, which has indirect regulation on downstream regulators of terminal chondrocyte differentiation (Lepetsos et al., 2019). In OA, the chondrocytes shift to a degradative phenotype where the NF-κB transcription factors elicit the secretion of many degradative enzymes, such as MMPs and ADAMTS, which lead to articular cartilage breakdown (Rigoglou and Papavassiliou, 2013). HIF-1α has an anti-catabolic function in the maintenance of articular cartilage through suppression of NF-κB signaling (Okada et al., 2020). In addition, NF-κB enhances HIF-1α expression to expand HIF-1α regulatory potential, which leads to the more effective execution of the host-defense response. In turn, NF-κB promotes HIF-1α activation during hypoxia expanding its prosurvival function (Rius et al., 2008). In the OA mouse model, Loganin ameliorates cartilage degeneration and attenuates subchondral bone remodeling through inhibition of NF-κB activity in chondrocytes to delay OA development (Hu et al., 2020a). Qigu capsule, by upregulating the NF-κB/HIF-1α signaling pathway, promotes bone formation and osteocyte autophagy (Wang et al., 2021). Moreover, Icariin can increase chondrocyte vitality by promoting HIF-1α expression and anaerobic glycolysis (Wang et al., 2020b). Thus, in an oxygen-deficient environment, through NF-κB signaling pathway to activate the potential of HIF-1α is a critical way to delay the development of OA.

### 5.4 PI3K/AKT/mTOR

PI3K/AKT/mTOR, an important and complex signaling pathway, is related to the initiation and progression of various diseases such as cancer, diabetes, and cardiovascular diseases (Sun et al., 2020). HIF-1α is a known critical regulator of glucose metabolism, positively regulated by AKT/mTOR (Liu et al., 2021). Suppressing the PI3K/AKT/mTOR/4E-BP pathway could be served to regulate HIF-1α expression at the translational step (Sun et al., 2007). By increasing HIF-1α translation, the activation of the PI3K/mTOR signaling pathway could increase HIF-1α protein levels without altering HIF-1α mRNA levels (Karar and Maity, 2011). Delphinidin can specifically decrease the CoCl_2_ and epidermal growth factor-induced HIF-1α protein expression by blocking the PI3K/AKT/mTOR/p70S6K signaling pathways (Kim et al., 2017). Inhibition of the PI3K/AKT/mTOR signaling pathway could promote the autophagy of articular chondrocytes and attenuate inflammation response in rats with OA (Xue et al., 2017). In human chondrocytes, the PI3K/AKT/mTOR axis could inhibit apoptosis and enhancement autophagy, then delay the progression of OA (Cai et al., 2019). The dual inhibition of PI3K and mTOR could be a promising approach to inhibit the PI3K/Akt pathway than inhibition PI3K alone, and which could produce better treatment outcome (Chen et al., 2013). Sun et al. (2018) found that LncHIFCAR, *via* significantly inhibiting hypoxia-induced activation of PI3K/AKT/mTOR pathway, positively upregulates HIF-1α, VEGF, and BNIP3 inducing inflammatory response, matrix synthesis, apoptosis to improve cell injury and promote OA development. In a word, PI3K/AKT/mTOR signaling pathway plays a significant role in the pathogenesis of OA, which is an essential pathway for targeted treatment of OA.

### 5.5 Runx2

Runx2 belongs to the Runx family, which could regulate DNA transcription (Komori, 2003). Runx2 is essential for osteoblast differentiation and chondrocyte maturation (Vimalraj et al., 2015). It could upregulate the expression of MMP-9, MMP-13, and VEGF (Komori, 2018), and enhance chondrosarcoma cells (Sun et al., 2009). Furthermore, Runx2-deficient mice lack osteoblast, bone formation, and chondrocyte maturation is markedly inhibited (Komori, 2019). Moreover, Runx2 heterozygous knockout mice exhibit loss of vascularization and VEGF expression in hypertrophic chondrocytes, resulting in loss of endochondral ossification (Lee et al., 2017). In bone tissue, Runx2 can stabilize the HIF-1α structure via the inhibition of HIF-1α ubiquitination in order to promote angiogenesis in growth plate hypertrophic chondrocytes (Xu, 2018). N-acetyl cysteine can attenuate hypoxia-mediated upregulation of HIF-1α, Runx2, and osteocalcin protein expressions, as well as inhibit extracellular matrix calcification (Balogh et al., 2019). HIF-1α can reduce the expression of the crucial osteogenic gene Runx2 in normoxia (Liu et al., 2019). Through the transcriptional regulation of Runx2, HIF-1α plays an important role in self-repair of the glycolytic metabolism of degenerative chondrocytes (Kong et al., 2021). Runx2 is not essential for the HIF-1α response, but Runx2 functions together with HIF-1α using sites within the Runx2 RUNT domain to stimulate angiogenic gene expression in bone cells, and induce VEGF gene expression (Kwon et al., 2011). In addition, Lee et al. (2012a) found that Runx2 stabilizes HIF-1α through binding to the ODDD to block the interaction between pVHL and HIF-1α, stimulating angiogenesis in growth plate hypertrophic chondrocytes.

In clinical and lab experiments, doctors and scientists have explored the therapeutic mechanism of the HIF-1α protein. The present studies found that a large number of treatments for OA are related to HIF-1α protein, such as hyaluronic acid, pentosan polysulfate, Mg^2+^, FBW7, mechanical stimulation, MicroRNA, Circular RNAs, and Chinese Herbal Medicine. Each kind of treatment has its corresponding therapeutic mechanism, target tissue or cells, and special therapeutic effect on OA, as shown in Table 1.

**TABLE 1:** Regulation of HIF-1α as potential therapeutic options for OA.

Regulators	Tissue (cell) type	Effects	Reference
Hyaluronic acid	Rat articular cartilage	Potentiates the synthesis of ECM, promotes the redifferentiation of rat articular chondrocytes and the synthesis of hyaline cartilaginous ECM	Ichimaru et al. (2016)
Pentosan polysulfate	Dog articular chondrocytes	Inhibits IL-1β-induced iNOS, c-Jun, and HIF-1α mRNA upregulation, inhibits IL-1β + TNF-α + LPS-induced iNOS protein expression	Bwalya et al. (2017b)
Mg^2+^	Human cartilage and synovium explants, rat articular cartilage	Reduces the expression of IL-6 and MMP-13, enhances the expression of HIF-1α and SOX-9, reduces the expression of NF-κB, promotes cartilage matrix synthesis, suppresses synovial inflammation	Yao et al. (2019)
Mg^2+^ and Vitamin C	Mice articular cartilage	Alleviate structural degeneration, prevent cartilage degeneration, promote cartilage matrix synthesis, suppress the expression of IL-6 and MMP-13, alleviate pain-related animal behaviors, ameliorate inflammation-induced pain, inhibit osteophytes formation	Yao et al. (2021)
FBW7	Human articular cartilage	Contributes to the production of collagen II, aggrecan, and SOX-9, inhibits the expression of collagen I and Runx-2, and protects the chondrocyte viability	Zhu et al. (2020)
Mechanical stimulation	Rat articular cartilage	Decreases aggrecan and ADAMTS-5, control cartilage homeostasis	Shimomura et al. (2021)
miR-144-3p	Human chondrocyte	Increases mitochondrial fusion	Song et al. (2017)
miRNA-411	Human chondrocyte	Promotes chondrocyte autophagy	Yang et al. (2020)
CircRNA-UBE2G1	Human normal chondrocyte	Reduces the expression of pro-inflammatory cytokines (IL-1β, IL-6, and TNF-α)	Chen et al. (2020)
Agnuside	Rat articular cartilage	Relieves the state of hypoxia, alleviates synovitis and fibrosis	Zhang et al. (2021b)
Casticin	Rat articular cartilage	Improves hypoxia and inflammation in synovium tissue, as well the synovial fibrosis in rats	Li et al. (2020b)
Vitexin	Human cartilage tissue	Inhibits PGE2 and NO production, inhibits HIF-1α expression, inhibits inflammatory responses	Yang et al. (2019a)
Baicalin	Mouse articular cartilage	Promotes ECM synthesis and marker genes (SOX-9, aggrecan, collagen II, MMP-9, MMP-13, ADAMTS-5) expression in chondrocytes	Wang et al. (2020c)
Icariin	Murine chondrocyte	Inhibits PHD activity, increases chondrocyte proliferation, differentiation, and integration with subchondral bone formation, promotes articular cartilage repairment	Wang et al. (2016)
Icariin	Mice chondrocyte	Increases chondrocyte ECM synthesis, maintains chondrocyte morphology, promotes anaerobic glycolysis metabolism	Wang et al. (2020b)

ADAMTS, a disintegrin and a metalloprotease with thrombospondin motifs; ECM, extracellular matrix; LPS, lipopolysaccharide; IL, interleukin; iNOS, inducible nitric oxide synthase; MMP, matrix metalloproteinase; PGE2, prostaglandin E2; PHD, prolyl-hydroxylase; TNF-α, tumor necrosis factor-α.

## 6 The Treatments With HIF-1 α in Osteoarthritis

### 6.1 Hyaluronic Acid

Hyaluronic acid (HA), a non-sulfated glycosaminoglycan, is a major component of the extracellular matrix, which is involved in the effects of lubricating and cushioning properties and anti-inflammatory (Graca et al., 2020). HA could promote the recruitment of neutrophil cells, involved in the phagocytosis of the debris and removal of dead tissue, and the subsequent release of TNF-α, IL-1β, IL-8 (Tavianatou et al., 2019). It has been shown that by inhibiting MMP-13, ADAMTS5, COX-2, and NF-κB in IL-1 stimulated chondrocytes, HA minimized cartilage loss in the OA model (Jin et al., 2020). In the presence of both HA and hypoxia the expression of CD44 was increased (Zhang et al., 2021a), and CD44 activates hypoxia-inducible HIF-1α signaling via the ERK pathway (Ryu et al., 2018). Hypoxia potentiates the anabolic effects of exogenous hyaluronic acid by a mechanism in which HIF-1α positively regulates the expression of CD44, enhancing the binding affinity for exogenous HA of OA chondrocytes (Ichimaru et al., 2016).

### 6.2 Pentosan Polysulfate

Pentosan polysulfate is a semi-synthetic sulfated polysaccharide compound manufactured from beech-wood hemicellulose by sulfate esterification of the xylopyranose hydroxyl group (Ghosh, 1999; Kumagai et al., 2010). Pentosan polysulfate significantly enhanced chondrogenesis and proteoglycan deposition (Bwalya et al., 2017a). In the early stages of OA treatment, pentosan polysulfate could inhibit chondrocyte proliferation while promoting a chondrocyte phenotype. However, the proliferative activity of chondrocytes with subsequent phenotypic shift is short and productive in an essential extracellular matrix component is less (Akaraphutiporn et al., 2020). An open clinical trial showed that pentosan treatment in patients with mild knee OA seemed to improve clinical assessments and C2C level of cartilage metabolism (Kumagai et al., 2010). Bwalya et al. (2017b) first demonstrated that pentosan polysulfate is a novel inhibitor of IL-1β-induced iNOS, c-Jun, and HIF-1α mRNA upregulation and iNOS protein induction which could be beneficial for the prevention and treatment of OA.

### 6.3 Mg^2+^

Mg^2+^ has been widely used to treat tachyarrhythmia, preeclampsia, and orthopedic surgery (Yoshizawa et al., 2014; Huang et al., 2015). Recent studies have found that Magnesium deficiency was considered a major risk factor for OA development and progression (Li et al., 2016). Proteome analysis has also indicated that Mg-based biomaterials regulate proteins associated with cell chondrogenesis and cartilage formation, which is beneficial for cartilage regeneration (Martinez Sanchez et al., 2019). Yao et al. (2019) showed that Mg^2+^ could enhance HIF-1α and SOX-9 expression and reduce NF-κB expression in human cartilage tissue explant after IL-1β induction *in vitro*, promoting cartilage matrix synthesis and the suppression of synovial inflammation. A further study found that vitamin C could enhance the promotive effect of Mg^2+^ on HIF-1α expression in cartilage and alleviate joint destruction and pain in OA (Yao et al., 2021). In a word, Mg^2+^ may be a cost-effective alternative treatment for OA in the future.

### 6.4 FBW7

FBW7, a kind of tumor suppressor, is a member of the SKP1–cullin1–F-box protein complex and the most highly mutated F-box protein in human cancer (Hong et al., 2016). As an important regulator of endothelial functions, FBW7 can promote angiogenesis, leukocyte adhesion and protect the integrity of the endothelial barrier (Wang et al., 2013). FBW7 reciprocally regulated cell migration and angiogenesis in a HIF-1α dependent manner (Flugel et al., 2012). The combination of FBW7 and Usp28 could regulate Myc protein stability in response to DNA damage (Popov et al., 2007). After DNA damage and cellular differentiation, MDM2 and FBW7 can cooperate to regulate the levels of the pro-proliferative △Np63α protein (Galli et al., 2010). Zhu et al. showed that FBW7, through suppressing HIF-1α and decreasing VEGF level, produces collagen II, aggrecan, and SOX-9, inhibiting collagen I and Runx-2 expression and delaying the IL-1β induced chondrocytes degeneration (Zhu et al., 2020). Therefore, FBW7 interacts with HIF-1α and regulates VEGF, effective treatment for effectively delaying the process of OA.

### 6.5 Mechanical Stimulation

During the development of the skeletal, mechanical stimulation plays a very important role in bone metabolism (Shea and Murphy, 2021). The mechanical stress could be via gap junctions transmitted, then osteocytes will release signaling factors and activate the bone remodeling response (Liang et al., 2021). Barcik et al. (2021) demonstrate that optimizing the ratio between mechanical stimulation and resting could contribute to more robust fracture healing in the future. In cartilage, it has been established that mechanical stimulation can greatly enhance cellular metabolic activity affect the proteoglycan and collagen content (Zhang and Yao, 2021). An experiment found that tensile stress of chondrocytes through regulating HIF-1α under hypoxic conditions to maintain cartilage homeostasis and ameliorates articular cartilage degeneration (Shimomura et al., 2021).

### 6.6 MicroRNA

MicroRNA (miRNA) is a kind of non-coding RNA that is approximately 18–25 nucleotides long (Ko et al., 2020). The research found that miRNAs are important regulators of genes to cartilage degeneration, inflammation, proteolytic enzyme synthesis, autophagy, apoptosis, and signaling pathways (Cong et al., 2017). IL-1β controls the bioavailability of proteases responsible for OA cartilage degradation, and miR-144-3p mimic transfection could downregulate levels of IL-1β expression while blocking the MAPK, NF-κB, and PI3K/AKT signaling pathways relating to IL-1β production (Lin et al., 2021). Song et al. (2017) found that peroxisomal dysfunction will upregulate miR-144-3p. Knocking down HIF-1α in normal chondrocytes could suppress CRAT expression while stimulating miR-144-3p, which provides a therapeutic strategy for OA. MMP-13 as a direct target gene of miR-411 in chondrocytes, overexpression of miR-411 can inhibit the MMP-13 expression and increase the expression of type II collagen, type IV collagen expression chondrocytes (Wang et al., 2015). MiRNA-411, aberrantly expressed in articular cartilage, regulates chondrocyte autophagy in OA by targeting HIF-1α and identifying the related molecular mechanism (Yang et al., 2020). Thus, MicroRNAs are promising therapeutic strategies for treating OA through the HIF-1α signal pathway.

### 6.7 Circular RNA

Circular RNAs (CircRNAs), covalently closed endogenous RNAs, are noncoding RNAs characterized by a covalent loop configuration without 5′ end caps or 3′ poly(A) tails (Ni et al., 2021). Furthermore, circRNAs are involved in the pathophysiological process of various diseases, such as diabetes mellitus, neurological disorders, cardiovascular diseases, skeletal muscle disease, and cancer (Kristensen et al., 2019). CircRNA.33186 can significantly upregulate in IL-1β treated chondrocytes and cartilage tissues of a destabilized medial meniscus-induced OA mouse model (Zhou et al., 2019b). Down-regulation of CircRNA.0001723 can suppress HIF-1α protein expressions, autophagy, and the activation of HIF-1α significantly reduce TNF-α, IL-1β, IL-6, and IL-18 levels *in vitro* inflammation model (Li et al., 2020a). Chen et al. (2020) demonstrated that HIF-1α overexpression could restore the effects of circRNA-UBE2G1 downregulation on Lipopolysaccharide-induced chondrocytes injury. Therefore, CircRNA also could be a target for regulating OA.

### 6.8 Chinese Herbal Medicine

#### 6.8.1 Agnuside, Casticin, and Vitexin

Agnuside is a non-toxic iridoid glycoside isolated from the leaf extract of Vitex negundo (Pillarisetti and Myers, 2018), which has effects of antioxidation, anti-inflammatory, analgesia, and antioxidant (Ramakrishna et al., 2016; Zhang et al., 2021b). Agnuside has a significant anti-inflammatory activity against acute inflammation by modulating the host immune response in rats (Pandey et al., 2012). Zhang et al. (2021b) found that agnuside can alleviate fibrosis and synovitis in experimental knee OA through the inhibition of HIF-1α accumulation and NLRP3 inflammasome activation.

Casticin, a polymethyl flavone with a molecular formula of C19H18O8, is derived mainly from the Vitex species of the family Verbenaceae (Chan et al., 2018). The main pharmacological properties of casticin include anti-inflammatory, anticancer, anti-asthmatic, anti-angiogenic, immunomodulatory, analgesic activities, etc. (Chan et al., 2018). Chu et al. (2020) showed that casticin through preventing cartilage degradation, reduces the progression of OA. Li et al. (2020b) found that casticin can alleviate monoiodoacetic acid-induced knee OA by inhibiting HIF-1α/NLRP3 inflammasome activation. In addition, casticin could significantly inhibit IL-1β-induced NO and PGE2 production, and COX-2 and iNOS expression in human OA chondrocytes. It also suppressed the levels of TNF-α and IL-6 and decreased the production of MMP-3, MMP-13, ADAMTS-4, and ADAMTS-5 in IL-1β-stimulated chondrocytes (Mu et al., 2019).

Vitexin, a kind of flavonoid, has anti-oxidant, anti-inflammatory, and anti-neoplastic effects, which have potential treatment of cancer, cognitive deficits, depressant, analgesic, cardiac hypertrophy arthropathies, metabolic disorder, etc. (He et al., 2016). It is an effective inhibitor of HIF-1α (Sartori-Cintra et al., 2012). Xie et al. (2018) demonstrated that vitexin could inhibit both the extracellular matrix stress-activated NF-κB pathway and the decreased apoptosis. Finally, attenuated the progression of OA in rats (Xie et al., 2018). Yang et al. (2019a) showed that Vitexin would alleviate IL-1β-induced inflammatory responses in chondrocytes from OA patients, which may be attributed partly to the inhibition of HIF-1α pathway.

All in all, in animal experiments, Vitex species is an effective drug for the treatment of OA, which mainly by inhibiting HIF-1α, improving inflammation in chondrocytes to alleviate the progression of OA. They could be considered as an effective drug for the treatment of OA in the future.

#### 6.8.2 Baicalin

Baicalin, a flavonoid extracted from Scutellaria baicalensis Georgi, with the effects of antibacterial, diuretic, anti-inflammatory, anti-metamorphosis, and antispasmodic (Yang et al., 2018). Through upregulating the autophagy markers, Beclin-1 expression increased autophagy. Baicalin promoted autophagic flux, decreased the apoptosis rate induced by IL-1β, upregulated anti-apoptotic Bcl-2 expression, and inhibited the extracellular matrix degradation (Li et al., 2020c). Baicalin can significantly elevate the mRNA levels of eNOS and inhibit oxidative stress in endplate chondrocytes (Pan et al., 2017b). Research showed that Baicalin could relieve cell apoptosis aroused by hypoxia might be achieved through activating Nrf2/HO-1-mediated HIF-1α/BNIP3 pathway (Yu et al., 2019). In addition, by activating the HIF-1α/SOX-9 pathway, Baicalin promoted extracellular matrix synthesis and had a protective effect on mouse chondrocytes *in vitro* (Wang et al., 2020c). Therefore, Baicalin can increase autophagy, reduce chondrocyte apoptosis caused by hypoxia and delay the progression of OA.

#### 6.8.3 Icariin

Icariin, a typical flavonoid compound extracted from Epimedium, has bone-protective, anticancer, and anti-inflammatory effects (Zu et al., 2019; Wang et al., 2020a). The study showed that Icariin is a safe and strong chondrocyte anabolic agent that could affect the proliferation of chondrocytes and reduce the degradation of the extracellular matrix (Li et al., 2012). Icariin can inhibit cell migration and proliferation, thus preventing the expression of cytokine IL-1β. Which further reduces the inflammatory response, such as reducing the MMP-14 expression level and inhibiting the endoplasmic reticulum stress (Pan et al., 2017a). In recent years, researchers gradually found that Icariin can relieve cell damage by promoting cell proliferation and suppressing apoptosis in oxygen-glucose deprivation-induced cells (Mo et al., 2017). Through competition for cellular iron ions, Icariin may likely inhibit human proline hydroxylase activity. Which could activate HIF-1α, and promote articular cartilage repair through regulating chondrocyte proliferation, differentiation, and integration with subchondral bone formation (Wang et al., 2016). What’s more, Icariin has an effect on promoting bone marrow stromal cell migration via the activation of HIF-1α (Zhu et al., 2018). Wang et al. (2020b) found that Icariin could promote HIF-1α expression and increase the expression of glycolytic enzymes, which contribute to glucose transfer and anaerobic glycolysis, finally facilitating chondrocyte vitality and treating OA. In a word, Icariin can effectively increase glycolysis, promote the proliferation of chondrocytes, slow down inflammation, and reduce the expression of MMP-14 in the extracellular matrix.

## 7 Conclusion

Degenerative OA is one of the significant medical problems and social issues worldwide. However, there are no effective treatments to improve the clinical outcomes of patients with degenerative OA. Researchers have found that the articular cartilage existed in a long-term hypoxic environment, and the cartilage hypoxia of OA is more significant. HIF-1α is a kind of hypoxia transcription factor. Under hypoxia, stabilized HIF-1α accumulates in the cytoplasm and migrates to the nucleus, which will initiate transcription and upregulate its activity. More and more evidence suggests that HIF-1α is closely associated with the pathogenesis of OA, such as improving chondrocyte apoptosis and autophagy, regulating chondrocyte extracellular matrix environment, and regulating glycolysis and mitochondrial function. Therefore, further studies on the role of HIF-1α in OA and its potential mechanisms may provide new therapeutic strategies for the treatment of this disease.

Many treatments involving HIF-1α have been reported to have therapeutic effects for many cell and mice OA models. However, existing research of pharmaceutical and exact mechanisms is very scarce. To better understand the role of HIF-1α in degenerative OA, we need to further study the mechanisms of the HIF-1α in degenerative OA, whether cell or animal model experiments and several clinical trials of HIF-1α regulation, which is beneficial to translate these experimental data into clinical applications. Taken together, this review emphasizes the great potential of HIF-1α and hypoxia signal as a target for treatments of degenerative OA.

In the perspective of future research, a series of key issues regarding the role of HIF-1α in the treatment of degenerative OA still require to be answered, including how various agents regulate HIF-1α signaling pathways in homeostasis and how HIF-1α interacts with other signaling pathways. This is a key limitation for the medications that regulate inflammation and metabolism of the chondrocyte to treat degenerative OA. Ultimately, the detailed mechanisms for the therapies, including medicinal and non-medicinal therapeutic options, targeting the HIF-1α and hypoxia signaling in degenerative OA treatment, still need to be further explored. Meanwhile, founding and creating novel and reliable therapeutic methods with less or no side effects will be the next breakthrough.
